# Neurosurgical management of progressive adhesive arachnoiditis with ascending myelopathy following obstetric epidural Anesthesia: An illustrative case with ten-year follow-up and review of the literature

**DOI:** 10.1016/j.bas.2026.107494

**Published:** 2026-11-04

**Authors:** Michael Veldeman, Anke Hoellig, Beate Huffmann, Hans Clusmann

**Affiliations:** Department of Neurosurgery, RWTH Aachen University Hospital, Aachen, Germany

**Keywords:** Adhesive arachnoiditis, Spinal arachnoiditis, Epidural anesthesia, Ascending myelopathy, Syringomyelia

## Abstract

**Introduction:**

Chronic adhesive arachnoiditis (CAA) following obstetric neuraxial anesthesia is an exceptionally rare but potentially devastating complication. While the diagnosis and conservative management of post-epidural CAA have been described, the neurosurgical management of patients developing progressive ascending myelopathy over many years remains virtually undocumented.

**Research question:**

What is the long-term neurosurgical decision-making process, and what are the outcomes, in a patient with post-epidural CAA developing progressive ascending myelopathy to the brainstem over a decade?

**Material and methods:**

We report a single illustrative case with ten-year prospective follow-up and conducted a structured PubMed/MEDLINE search to identify previously published cases of post-neuraxial anesthesia CAA with ascending, holospinal, or surgically managed disease.

**Results:**

A woman in her mid-twenties developed CAA following obstetric epidural analgesia complicated by postpartum meningitis. Over ten years she underwent eighteen neurosurgical procedures, including ventriculoperitoneal shunting, hemilaminectomy with ventral arachnoid cyst decompression, cystoperitoneal shunting, suboccipital decompression with C1 arch resection, Rickham reservoir implantation, and integration of fourth ventricular and cervical syrinx drainage into a dual-valve shunt system. Progressive ascending myelopathy ultimately reached the brainstem, necessitating tracheostomy and intermittent mechanical ventilation. Repeated short courses of high-dose corticosteroids were used adjunctively for acute exacerbations. Despite near-complete tetraplegia, the patient remained fully cognitively intact at ten-year follow-up – the longest reported for surgically managed post-epidural CAA.

**Discussion and conclusion:**

Comparison with previously published cases suggests this represents the first documented progression of post-epidural CAA to permanent respiratory failure requiring tracheostomy; earlier reports describe brainstem involvement but not this degree of respiratory compromise. Stepwise neurosurgical intervention appeared to provide periods of stabilization, but could not halt the underlying fibrotic process. The goal of surgery in this disease is best framed as stabilization rather than cure. Clinicians should counsel patients that long-term progression cannot be excluded, and that surveillance imaging of the full neuraxis is warranted indefinitely.

## Highlights

• Post-epidural chronic adhesive arachnoiditis can ascend from lumbar to brainstem.
• We report the longest documented follow-up of surgically managed post-epidural CAA.
• StepWise neurosurgical intervention may delay but cannot halt CAA progression.
• Dual-valve shunt systems can address multi-compartment CSF pressure imbalance.
• Long-term full-neuraxis MRI surveillance may be warranted in post-epidural CAA.

## 1 Introduction

Chronic adhesive arachnoiditis (CAA) is an extremely rare condition characterized by progressive inflammation of the arachnoid membrane leading to fibrosis, adhesion formation, and ultimately encapsulation and ischemic atrophy of intrathecal neural elements (Rice et al., 2004). First described as a distinct disease entity by Victor Horsley in 1909, fewer than 1000 cases worldwide, have been reported in the fifty years preceding a comprehensive 2004 review (Rice et al., 2004). The condition is most commonly encountered following contrast myelography with oil-based agents, repeated spinal surgery, or central nervous system infection; neuraxial anesthesia represents a rare but recognized trigger (Rice et al., 2004; Killeen et al., 2012).

The pathological sequence was described by Burton (1978) as progressing from radiculitis, pia-arachnoid inflammation with nerve root swelling and hyperemia, through arachnoiditis with collagen deposition, to adhesive arachnoiditis with dense fibrosis, encapsulation of nerve roots, and progressive atrophy secondary to vascular compromise. When this process occurs adjacent to the spinal cord, disrupted flow of cerebrospinal fluid (CSF) generates a pressure gradient that drives interstitial seepage of CSF into the cord, producing syringomyelia (Killeen et al., 2012).

Published case reports of CAA following obstetric neuraxial anesthesia have appeared sporadically since the 1980s (Killeen et al., 2012; Boiardi et al., 1983; Na et al., 2011; AlMutairi et al., 2024). These reports, predominantly in anesthesiologic journals, focus on the causative agent, diagnosis by MRI, and conservative management, principally corticosteroids. Neurosurgical management has been documented only in the most severely affected cases and invariably as a brief component of anesthesiologic case reports, with follow-up rarely exceeding two years (Killeen et al., 2012; Boiardi et al., 1983).

We report a patient who developed CAA following obstetric epidural anesthesia and whose disease pursued an unrelentingly ascending course over ten years, requiring eighteen neurosurgical procedures. Based on a structured literature search (Methods), this represents the longest documented follow-up of surgically managed post-epidural CAA and, to our knowledge, the first case to document progression to permanent respiratory failure requiring tracheostomy. Our aim is to provide a detailed account of the neurosurgical decision-making at each stage of this disease, including the clinical rationale for offering repeated surgical intervention rather than conservative surveillance alone.

## 2 Methods

Clinical data were extracted from the patient's discharge letters and operative reports held at our institution. All time points are expressed relative to the index event (epidural anesthesia and vaginal delivery) rather than as calendar dates, to preserve anonymity.

A structured literature search of PubMed/MEDLINE was performed in April 2026 using combinations of the terms "arachnoiditis," "adhesive," "chemical," "epidural," "spinal an(a)esthesia," and "ascending." Records were screened for reports of chronic adhesive arachnoiditis following neuraxial (epidural or spinal) anesthesia. Cases with an ascending or holospinal disease pattern and/or surgical management were included in the structured comparison (Table 2). This search was restricted to a single database without a formal PRISMA methodology, and should therefore be regarded as a structured but non-exhaustive literature comparison rather than a systematic review in the strict sense. The reference lists of all identified reports were additionally screened for further relevant citations.

## 3 Illustrative case

Throughout this ten-year course, the rationale for offering surgical intervention rather than continued conservative surveillance was consistent: each procedure was undertaken in response to new or progressive neurological deficit that correlated with a new, structurally defined and potentially remediable CSF compartment or obstruction on imaging (a cyst, a syrinx, ventricular dilation), rather than as a prophylactic or scheduled step. Consistent with the CSF-flow-obstruction model outlined above, the treating team's working hypothesis was that relieving these mechanical obstructions could attenuate the pace of neurological decline, even though the underlying fibrotic process itself could not be halted. This rationale is revisited at each stage below and examined further in the Discussion.

### 3.1 Index event and initial presentation (year 1)

A woman in her mid-twenties with no relevant medical history underwent epidural analgesia for vaginal delivery. At the time of catheter placement, she reported immediate shooting pain radiating to both legs. Leg function did not fully recover after the block wore off. Within days she developed suspected bacterial meningitis, treated with intravenous vancomycin and meropenem at the referring hospital. The specific substance(s) administered epidurally were never established. Her son had been born at the time of this index event; his care would remain central to the family's and treating team's approach to each subsequent surgical decision over the following decade.

Two months later, she presented to our department with progressive headache, apathy, and nausea. Imaging demonstrated marked communicating hydrocephalus (Fig. 1A). A spinal MRI showed holospinal arachnoid adhesions (Fig. 1B–C). An emergency right frontal external ventricular drainage (EVD) was placed after which the patient rapidly recovered consciousness. After confirmed sterile CSF, a ventriculoperitoneal (VP) shunt was implanted (Codman Hakim Medos valve, 140 mmH_2_O). She was discharged in a state where she was mobile with the help of a walking frame. Neurophysiology demonstrated afferent disturbances in both arms and the right leg consistent with myelopathy.

**Fig. 1:**
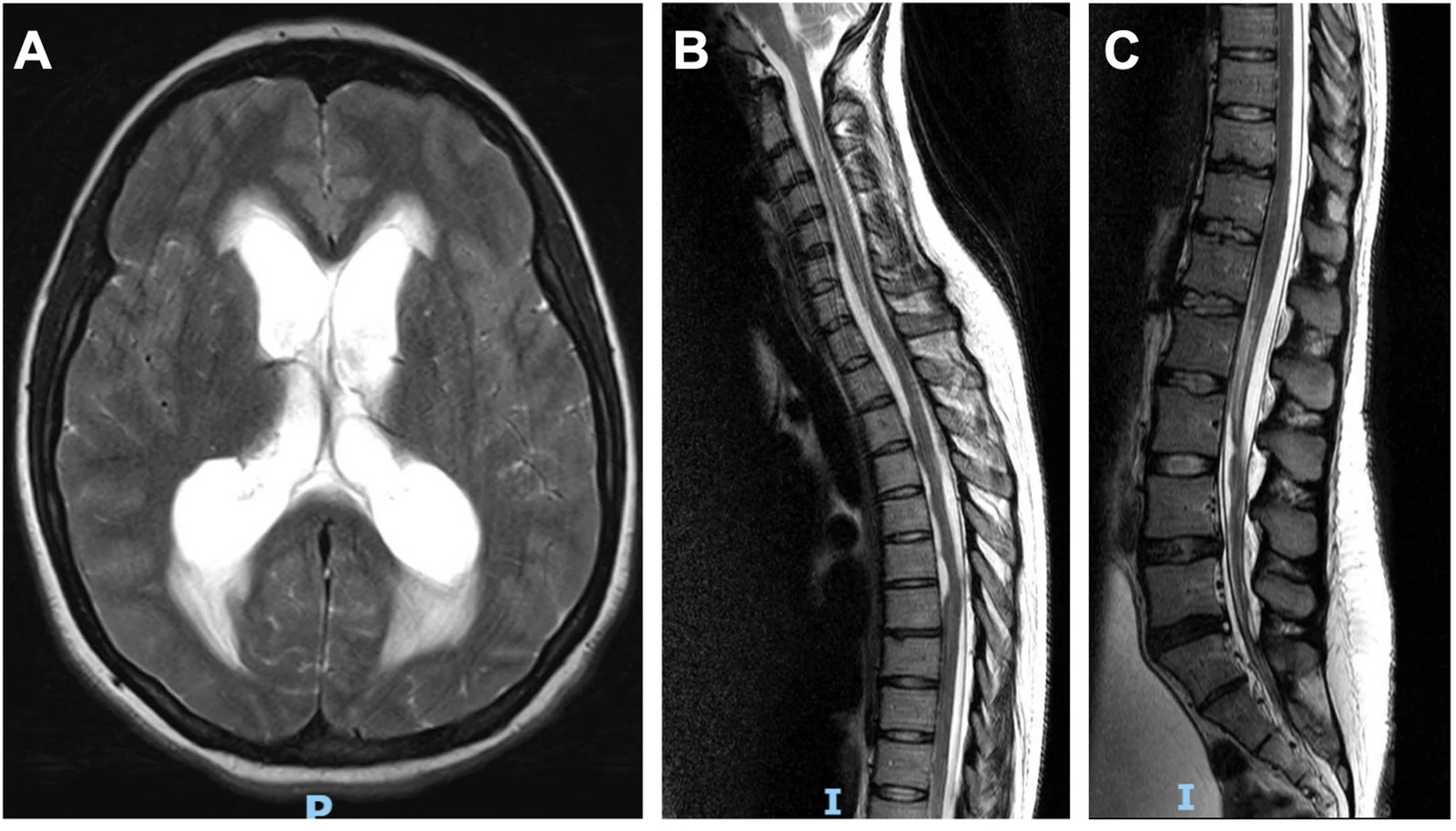
Initial neuroimaging at presentation (Year 1, month 2). (A) Axial T2-weighted MRI of the brain demonstrating marked dilatation of the lateral and third ventricles with periventricular T2 signal hyperintensity consistent with transependymal CSF seepage, indicative of communicating hydrocephalus. **(B)** Sagittal T2-weighted MRI of the cervicothoracic spine demonstrating early ventral adhesions at the level of Th4–6, with further tethering of the spinal cord to the ventral dural lining. **(C)** Sagittal T2-weighted MRI of the thoracolumbar spine demonstrating diffuse ventral adhesion of the spinal cord and conus to the anterior thecal sac throughout the lower thoracic and upper lumbar segments. Panels B and C represent the first imaging confirmation of spinal adhesive arachnoiditis extending to the mid-thoracic level at the time of initial neurosurgical presentation.

### 3.2 Acute paraparesis and ventral arachnoid cyst (month 3)

Seven days after discharge, she represented with rapidly progressive paraparesis. Emergency MRI demonstrated a space-occupying ventral CSF collection at the sixth-to-seventh thoracic vertebral level (Th6/7) with myelopathy signal extending to the conus. To decompress this collection and relieve the acute paraparesis, she underwent hemilaminectomy at the eighth-to-ninth thoracic level (Th8/9) on the right and Th6/7 on the left. Intraoperatively the subdural space was completely obliterated caudally with no CSF flow; at Th6/7 a pressurized ventral arachnoid cyst was entered and decompressed. Dural and arachnoidal biopsies remained sterile. Postoperatively, her left leg function improved (medical research council (MRC) 3/5) but the right leg remained paralytic and by the end of the first year, she required a wheelchair. Thirteen days later, as a separate, staged procedure, a cystoperitoneal (CP) shunt was placed at Th6/7 to provide durable drainage of the ventral arachnoidal cyst compartment to the peritoneum (see Fig. 2A and C).

**Fig. 2:**
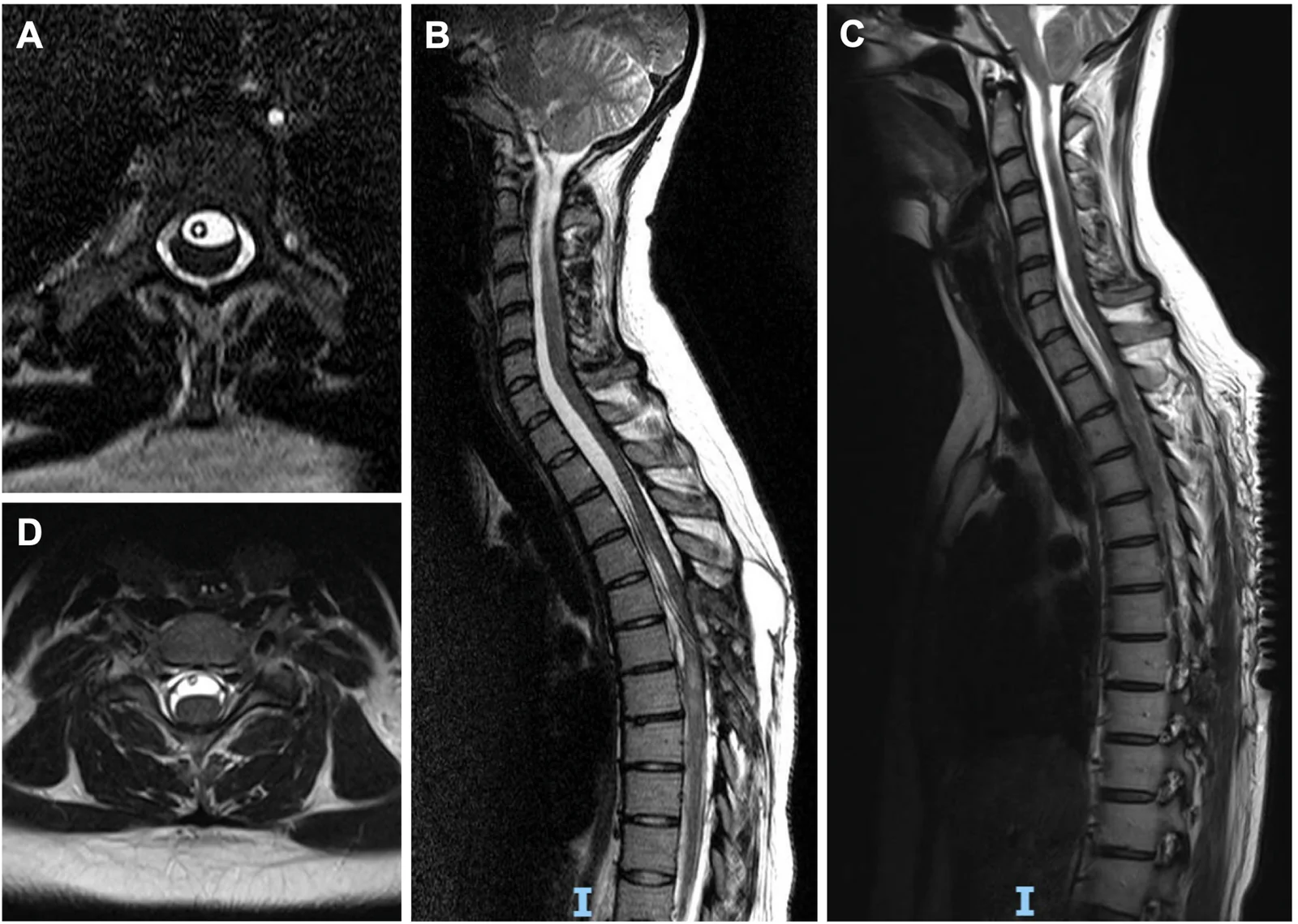
Catheter positions within ventral arachnoidal cyst compartments at two spinal levels (Year 1 month 3 and Year 5). (A) Axial T2-weighted MRI at the mid-thoracic level demonstrating the cystoperitoneal shunt catheter seated within the ventral arachnoidal cyst compartment, with the spinal cord displaced posteriorly. **(B)** Sagittal T2-weighted MRI of the cervicothoracic spine demonstrating the cranio-cervical cyst catheter coursing ventral to the spinal cord at the level of the cranio-cervical junction, draining the rostral extension of the compartmentalized arachnoidal disease. **(C)** Sagittal T2-weighted MRI of the thoracic spine showing the thoracic cystoperitoneal catheter within the ventral CSF collection at Th6/7. Note the absence of intramedullary signal change at this timepoint, consistent with a pre-syrinx stage. **(D)** Axial T2-weighted MRI at the cervical level showing ventral placement of the cranio-cervical catheter anterior to the cord. In both instances the catheter targets a discrete, compartmentalized ventral CSF collection rather than an intramedullary cavity, reflecting the pre-syrinx stage of disease at the respective timepoints.

### 3.3 Year 2

The following year the drainage system required three further interventions: complete exchange for insufficiency, repeat revision via hemilaminectomy Th4 for catheter dislocation as well as the addition of a second valve (Medos Hakim, 200 mmH_2_O, left flank) for symptomatic intracranial hypotension (to prevent overdrainage). By the end of the second year, persistent intracranial hypotension necessitated ligation of the CP shunt distal to the valve. She also underwent intercurrent evaluation for dysphagia, which at that time was attributed to esophageal dysmotility (in an achalasia-like pattern). In retrospect this might have represented early neurological involvement due to ascending, MRI occult, myelopathy.

### 3.4 Year 3 - 7

In year 4, a Rickham reservoir was implanted at the proximal end of the existing thoracic catheter, with distal ligation, to permit percutaneous access to the ventral CSF compartment. An immediate postoperative CSF fistula from the wound required emergency reconnection and fascial fixation of the reservoir the following day.

Outpatient surveillance demonstrated progressive enlargement of a ventral cervical cyst and dilatation of the 4th ventricle, tonsillar descent, and the development of a cervical syrinx. Fine motor disturbance of both hands had appeared. Therefore, a suboccipital decompression including C1 arch resection, duraplasty, adhesiolysis, 4th ventricle opening, and implantation of a cranio-cervical cyst catheter were performed (see Fig. 2B and D). The ventral cervical cyst collapsed progressively.

Around the beginning of year 7, progressive cervical syrinx and 4th ventricular dilation accompanied by onset of progressive hand weakness prompted a two-stage surgical intervention (see Fig. 3A). A re-adhesiolysis, opening of the 4th ventricle, Obex and opening of the cervical cord cysts was performed. Catheters were placed within the syrinx and 4th ventricle and connected to the existing Rickham reservoir (see Fig. 3B). Later on, the upper catheters were shortened and connected to the existing shunt system (see Fig. 3C). By the mid-year 7, her arm paresis partially recovered.

**Fig. 3:**
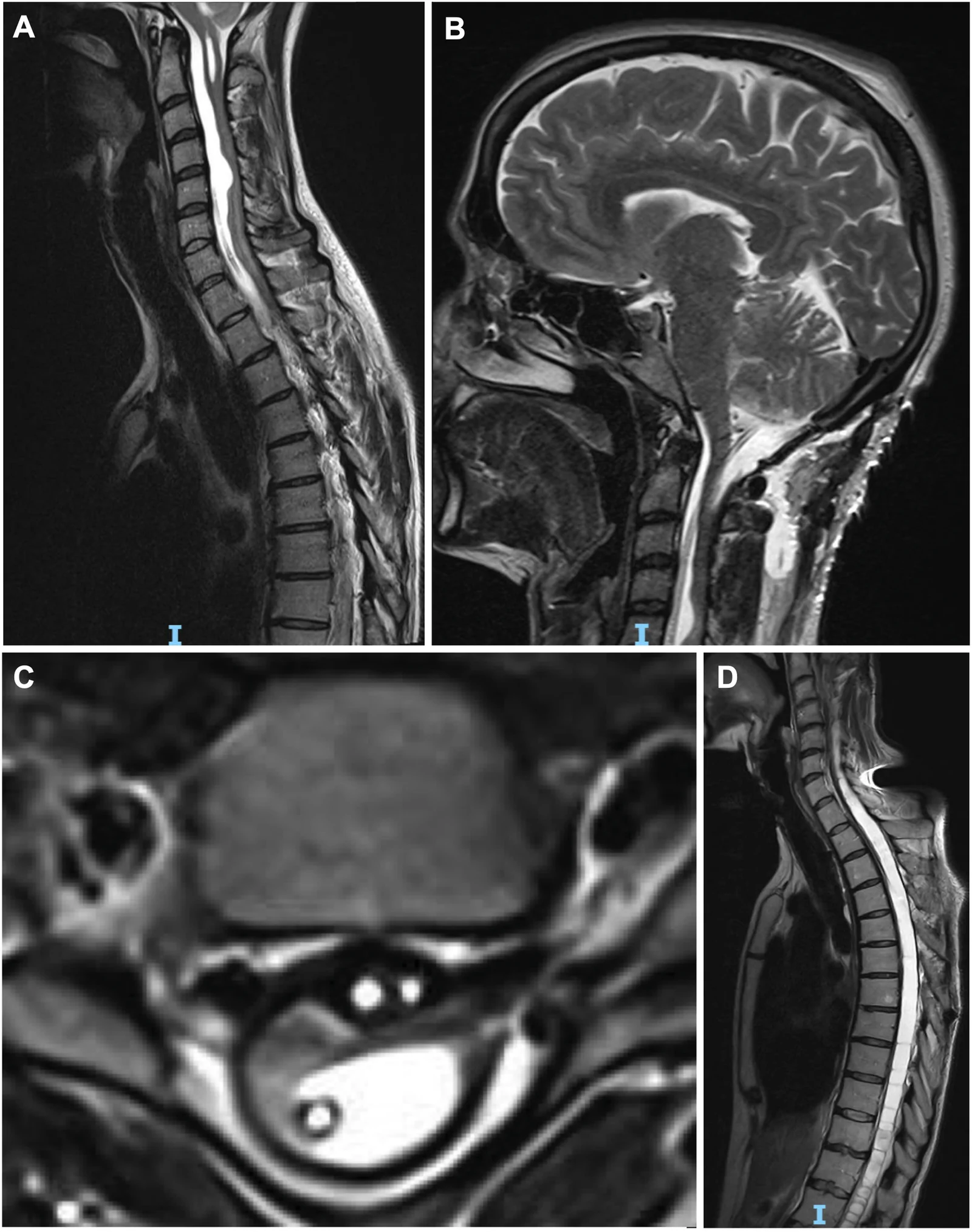
Disease progression from cervical syrinx formation to end-stage ascending myelopathy (Year 5 to Year 10). (A) Sagittal T2-weighted MRI of the cervicothoracic spine (Year 5, pre-operative) demonstrating intramedullary T2 signal hyperintensity and cavitation within the upper cervical cord extending into the medulla oblongata, consistent with syringomyelia complicating progressive adhesive arachnoiditis. Caudal displacement of the cerebellar tonsils was confirmed on parasagittal sequences. **(B)** Sagittal T2-weighted MRI of the cranio-cervical junction (Year 5, post-operative) following suboccipital decompression, C1 arch resection, duraplasty, and adhesiolysis. A catheter is visible coursing into the cervical syrinx. Decompression of the cranio-cervical junction has been achieved. **(C)** Axial T2-weighted MRI (Year 7) demonstrating three catheters within the spinal canal: one seated within the ventral thoracic arachnoidal cyst compartment, one within the ventral cervical compartment, and one within the intramedullary syrinx cavity, reflecting the definitive multi-compartment drainage configuration. **(D)** Sagittal T2-weighted MRI of the cervicothoracic spine (Year 9–10, most recent available imaging) demonstrating diffuse intramedullary T2 signal hyperintensity and expansion of the cervical and upper thoracic cord, consistent with maximal advanced syringomyelia. This image corresponds to the patient's clinical state of near-complete tetraplegia with intermittent ventilator dependence.

During rehabilitation later that year, the patient developed acute brainstem symptoms with bilateral high-grade facial palsy (House-Brackmann 4/5), complex oculomotor paresis with multidirectional nystagmus, bulbar dysarthria, dysphagia, and bilateral facial hypesthesia. This represented the first clinically overt brainstem involvement with MRI revealing ascending myelopathy reaching the medulla oblongata and pons. Phoniatric evaluation diagnosed bilateral vocal cord palsy and indicated the need for percutaneous endoscopic gastrostomy (PEG), which was placed by the summer of year 7.

Later that year, the shunt system was revised with separation into two independent drainage limbs: a frontal Certas Plus valve at pressure level 5 and a suboccipital Certas Plus valve at level 2, both draining via Y-connector to a single peritoneal catheter. The 4th ventricle regressed in size on post-operative MRI. The syrinx catheter was revised and reconnected to the posterior fossa shunt. Slow improvement of dysphonia and facial paresis was documented.

### 3.5 Year 8 - 10

The following years, progressive bilateral arm weakness developed, and the patient could barely self-feed. She eventually presented further with progressive dysarthria, reporting her voice had become increasingly quiet as a clinical indicator of impending respiratory compromise. An iatrogenic cerebellar hemorrhage was caused during aspiration of the Rickham reservoir which necessitated emergency suboccipital craniotomy with hematoma evacuation and EVD placement. Despite recovery of consciousness, the patient demonstrated absent cough and gag reflexes with profound dysphagia impeding extubation. Surgical tracheostomy was performed by the end of year 10, following family discussions in which the patient, communicating non-verbally, expressed consent.

### 3.6 Adjunctive medical management

Alongside these surgical interventions, the patient received repeated short courses of high-dose corticosteroids for acute neurological deterioration, particularly during episodes of worsening cervical myelopathy and, later, brainstem edema. The typical regimen was an initial bolus of 40 mg intravenous dexamethasone, followed by 4 mg four times daily with rapid tapering to minimize cumulative steroid exposure; the precise timing and duration of individual courses could not be reliably reconstructed from the clinical record. Recognized steroid-related side effects, including sleep disturbance and acne, occurred repeatedly and were a further reason for early weaning. Physiotherapy and rehabilitative care were integrated between surgical admissions throughout the disease course.

### 3.7 Current status

MRI more than ten years after the index event demonstrated broadly stable adhesive arachnoiditis with a minimal increase in 4th ventricular diameter and progression of syringomyelia (Fig. 3D). The patient carries a tracheostomy with speaking valve and requires intermittent mechanical ventilation. She has near-complete tetraplegia (wheelchair-dependent with support) and is PEG-fed. Her shunt system remains in situ with frontal valve at pressure level 5 and suboccipital valve at level 2. The treating team found no indication for further surgical intervention. Throughout this decade, the patient has remained fully cognitively intact and oriented. Her principal motivation to continue treatment has been the presence of her son, born at the time of her index event, who is now ten years old.

The complete chronological record of surgical interventions, indications, and immediate outcomes is presented in Table 1.

**Table 1:** Chronological record of neurosurgical interventions.

Time point	Procedure	Indication	Immediate outcome
Month 2 (Year 1)	Right frontal external ventricular drainage (EVD)	Symptomatic communicating hydrocephalus; obtunded patient	Rapid clinical improvement; consciousness restored
Month 2 (Year 1)	VP shunt, right frontal (Codman Hakim Medos valve, 140 mmH_2_O)	Definitive CSF diversion for post-infectious communicating hydrocephalus	Good recovery; mobile with walking frame at discharge
Month 3 (Year 1)	Hemilaminectomy Th8/9 right + Th6/7 left; decompression of ventral arachnoid cyst	Acute progressive paraparesis; space-occupying ventral CSF collection at Th6/7 with myelopathy signal to conus	Decompression confirmed on MRI; left leg MRC 3/5; right leg near-plegic
Month 3 (Year 1)	Cystoperitoneal (CP) shunt, level Th6/7 left	Ventral arachnoid cyst compressing spinal cord; to drain ventral CSF compartment to peritoneum	Cyst decompressed
Year 2	Complete CP shunt exchange	Shunt insufficiency	Drainage restored
Year 2	CP shunt revision via hemilaminectomy Th4	Catheter dislocation	Catheter repositioned
Year 2	Additional valve (Medos Hakim, 200 mmH_2_O, left flank)	Symptomatic intracranial hypotension from overdrainage	Underpressure symptoms resolved
Year 2	CP shunt ligation distal to valve	Persistent intracranial hypotension	Hypotension symptoms resolved; patient wheelchair-mobile
Year 4 (Month ∼32)	Rickham reservoir implanted at proximal thoracic catheter end + distal ligation	Growing ventral cervical CSF collection with cord compression at C1/2	Planned procedure
Year 4 (Month ∼32)	Emergency reconnection of Rickham reservoir; fixation to fascia	CSF fistula from wound due to disconnection	Fistula closed; no new neurological deficit
Year 5 (Month ∼51)	Suboccipital decompression + C1 arch resection + duraplasty + adhesiolysis + 4th ventricle opening + cranio-cervical cyst catheter	Enlarging ventral CCJ cyst causing arm symptoms; cervical syrinx formation; tonsillar descent	Improved hand paresthesias; cyst collapsed
Year 7 (Month ∼68)	Re-adhesiolysis + 4th ventricle + Obex + cervical cord cyst opening; catheter in syrinx and 4th ventricle → Rickham reservoir	Progressive cervical syrinx; 4th ventricle dilation; progressive hand weakness	Partial recovery of arm paresis
Year 7 (Month ∼69)	Catheter shortening + connection of 4th ventricle and syrinx to existing shunt system	Marginal catheter position in syrinx; persisting arm paresis	Further recovery of arm paresis
Year 7 (Month ∼70)	Shunt revision: dual drainage Frontal Certas Plus (level 5) + suboccipital Certas Plus (level 2); Y-connector to single peritoneal catheter	Acute brainstem syndrome; suspected shunt insufficiency and unfavorable pressure distribution between compartments	4th ventricle diameter regressed on MRI
Year 7 (Month ∼71)	PEG insertion	Bilateral vocal cord palsy; dysphagia; unsafe oral intake	Enteral feeding established
Year 7 (Month ∼71)	Syrinx catheter revision; coated catheter reimplanted; reconnection to posterior fossa shunt + duraplasty	Persistent brainstem symptoms; catheter position suboptimal	Slow improvement of dysphonia and bilateral facial paresis
Year 10 (Month ∼100)	Emergency cerebellar hematoma evacuation via suboccipital craniotomy + 4th ventricle EVD (iatrogenic hemorrhage during Rickham reservoir puncture attempt)	Acute cerebellar parenchymal hemorrhage with ventricular extension; Rickham puncture performed for suspected shunt dysfunction	Hematoma evacuated; patient stabilized; brainstem syndrome unchanged
Year 10 (Month ∼100)	Surgical tracheostomy	Progressive brainstem involvement with absent cough/gag reflex and profound dysphagia; inability to protect airway	Airway secured; intermittent mechanical ventilation commenced

Time points are expressed relative to the index event (epidural anesthesia and delivery). VP = ventriculoperitoneal; CP = cystoperitoneal; CCJ = cranio-cervical junction; EVD = external ventricular drain; PEG = percutaneous endoscopic gastrostomy; MRC = Medical Research Council; MRI = magnetic resonance imaging.

**Table 2:** Comparison of published cases of surgically managed post-neuraxial anesthesia adhesive arachnoiditis with ascending or holospinal disease.

Reference	Patient	Index procedure	Presenting features	Surgical management	Outcome	Follow-up
Boiardi et al., 1983 (Boiardi et al., 1983)	Case 3: 18M	Epidural (L2) bupivacaine 1% + epinephrine (orthopedic)	Progressive spastic paraparesis, urinary incontinence, hypesthesia to nipple level; then hydrocephalus, papilledema, CN VI + VII palsy	VP shunt only	Neurologically unchanged at 1 year	1 year
Killeen et al., 2012 (Killeen et al., 2012)	27F obstetric	Spinal: bupivacaine 0.5% + fentanyl (cesarean)	Communicating hydrocephalus 11 days postpartum; then rapid progressive paraplegia at 3 months	VP shunt; foramen magnum decompression + C1 laminectomy; laminectomy L5-S1 + T9; external syrinx drain; IV dexamethasone	Permanent paraplegia; sensory level T6/T10; wheelchair; suprapubic catheter	21 months
AlMutairi et al., 2024 (AlMutairi et al., 2024)	33F obstetric	Epidural (cesarean)	Severe holocephalic headache, nausea, vomiting weeks postpartum; communicating hydrocephalus; holospinal AA with CSF multiloculations to cervical level	VP shunt only	Stable at discharge; no reported neurological deficit	Discharge only
Present case 2015–2025	mid-twenties F obstetric	Epidural analgesia for vaginal delivery; complicated by postpartum meningitis	Hydrocephalus 2 months postpartum; acute paraparesis 7 days post-VP shunt; ascending myelopathy over 10 years to brainstem; tetraplegia; respiratory failure	VP shunt; hemilaminectomy ×2 + ventral cyst decompression; cystoperitoneal shunt + multiple revisions; Rickham reservoir; suboccipital decompression + C1 resection; dual shunt system (frontal + suboccipital Certas Plus); syrinx catheter; tracheostomy (see Table 1)	Near-complete tetraplegia; tracheostomy with intermittent ventilation; PEG-dependent; cognitively intact at 10 years	10 years

F = female; M = male; VP = ventriculoperitoneal; MRC = Medical Research Council; CN = cranial nerve; CCJ = cranio-cervical junction.

## 4 Discussion

Post-epidural CAA is among the rarest complications of neuraxial anesthesia. Rice et al. (2004) found fewer than ten published obstetric cases meeting diagnostic criteria in a comprehensive literature search, and large population surveys of up to 750,000 neuraxial anesthetics have identified no definite cases (Killeen et al., 2012). When it does occur, however, it can produce a devastating and relentlessly progressive course, as illustrated by the present case.

In comparison with previously published cases (Table 2), our patient's disease followed an unusually severe and prolonged trajectory. Boiardi et al. (1983) described the earliest documented case of post-epidural CAA complicated by hydrocephalus and cranial nerve involvement, a snapshot identical to our patient's early presentation. Their patient received a VP shunt and remained neurologically static at one year. Killeen et al. (2012) reported the closest structural parallel to our case: a young obstetric patient who underwent VP shunting, foramen magnum decompression, laminectomy, and a trial external syrinx drain over 21 months, ultimately remaining paraplegic. AlMutairi et al. (2024) described a 2024 obstetric case in which VP shunting alone appeared to arrest the disease at the hydrocephalic stage. Our patient's course extends the follow-up horizon dramatically beyond all published reports. Boiardi et al.'s Case 3 previously documented ascending disease reaching the cervicomedullary junction with cranial nerve involvement (Boiardi et al., 1983), but, to our knowledge based on the structured search described above, no prior report documents progression to respiratory failure requiring tracheostomy.

The etiology of our patient's CAA remains uncertain. The specific epidural injectate was never documented at the referring institution. Suspected postpartum bacterial meningitis, which preceded her arachnoiditis, may have been an independent or contributing trigger, or may have been the sole cause, with the epidural puncture serving as the portal of entry. Rice et al. (2004) noted that preservatives, vasoconstrictors, and blood products in the CSF are the most plausible chemical triggers, and emphasized the critical importance of meticulous record keeping. Killeen et al. (2012) raised the additional possibility of chlorhexidine contamination. The hyperergic (immune-mediated) mechanism proposed by Boiardi et al. (1983) remains the most plausible unifying framework when no contaminant can be identified.

The pathophysiological basis for ascending disease in our patient is CSF flow obstruction at successive levels. As adhesions formed and progressed cranially, trapped CSF under pressure produced compartmentalized cysts, then syringomyelia, and ultimately 4th ventricular obstruction and brainstem compression. Each neurosurgical intervention targeted a specific consequence of this process: the VP shunt addressed communicating hydrocephalus; the cystoperitoneal shunt drained the ventral thoracic cyst; the Rickham reservoir provided access to the cervical CSF compartment; the CCJ decompression addressed the tonsillar descent and cervical cyst; the dual shunt system attempted to balance drainage between the supratentorial and infratentorial compartments. The underlying fibrotic process continued regardless.

Several technical observations from this case deserve emphasis. First, the repeated failure of the cystoperitoneal shunt system, requiring five interventions in the first fourteen months, illustrates the particular challenge of draining a compartmentalized, fibrotic CSF space as opposed to a communicating hydrocephalus. The ventral arachnoidal cyst was not a simple fluid collection but a scarified, pressure-bearing compartment within a hostile inflammatory milieu. Second, the integration of 4th ventricular and syrinx drainage into a single dual-valve shunt system in 2021 represents an escalation of surgical complexity not previously described in this disease. The rationale to balance drainage between compartments with different pressure dynamics using independently adjustable valves was sound, but the long-term outcome could not be predicted. Third, this case illustrates that neurosurgical intervention in progressive CAA may slow the disease course but cannot halt the underlying fibrotic process. Each procedure addressed a specific, symptomatic consequence of ascending arachnoiditis, decompressing a cyst, draining a syrinx, re-establishing CSF pathways, and each appeared to provide a period of relative stabilization. Whether this translated into meaningful delay of the ultimate outcome cannot be established with certainty, but it is the considered judgement of the treating team that it did; no validated, serially applied outcome measure exists to substantiate this impression, and it should be read as clinical judgement rather than a measured or quantified effect of surgery. The goal of surgery in this disease is therefore best framed not as cure, but as buying time, time that, in this patient's case, has meant a decade of life with her child.

This framing has a direct implication for counselling. Post-epidural CAA most commonly affects young women of childbearing age, often at the very moment of becoming a mother. The prospect of progressive neurological deterioration, potentially over many years, through paraplegia, tetraplegia, and ultimately respiratory dependence, raises profound questions about quality of life that cannot be answered by the surgical team alone. Individual, repeated, and honest counselling, involving the patient, her family, palliative care specialists, and where appropriate a clinical ethicist, is an integral part of management from the earliest stages. This patient's decade-long perseverance in the face of severe disability was grounded in a clearly articulated personal goal. Not every patient will make the same choice, and that choice deserves the same respect.

## 5 Lessons

This case generates several lessons for neurosurgeons who may encounter patients with progressive CAA.

### 5.1 Surgical intervention can delay but may not prevent progression

Our team believes that the stepwise surgical approach, addressing each manifestation of disease as it arose, postponed the catastrophic neurological decline that might otherwise have occurred earlier. We cannot prove this. The disease was clearly advancing throughout the ten years regardless of surgical intervention. What surgery appeared to achieve was temporary stabilization at each stage, buying time rather than achieving cure. This honest uncertainty must be communicated to patients and families at each decision point.

### 5.2 Patient-centered goals of care must guide each decision

This patient has made repeated, informed decisions to proceed with surgery in the context of progressive disability. Her motivation, to remain alive and present for her son, has been explicit throughout. Every surgical decision in this case was made in partnership with the patient and her family, with full discussion of realistic expectations.

### 5.3 Ascending CAA represents a distinct and under-described clinical entity

The existing literature on post-epidural CAA describes predominantly lumbosacral or thoracolumbar disease. Our case, and Boiardi's Case 3 from 1983 (Boiardi et al., 1983) demonstrate that the inflammatory and fibrotic process can ascend to the cervicomedullary junction and beyond. Neurosurgeons managing these patients should anticipate this possibility and ensure that surveillance imaging covers the full neuraxis, including the posterior fossa and brainstem.

## Ethics declaration

This study was performed in compliance with relevant laws, regulatory frameworks and guidelines where the research took place. Ethics committee approval was not required under relevant laws and institutional guidelines. This is a case report. Consent of the patient involved was acquired. Ethical approval is waived.

This research follows the CARE guidelines and the CARE checklist.

## Patient consent

Written consent for publication was obtained from the patient's parents, who hold legal next-of-kin status. The patient is fully cognitively intact and was involved in all treatment decisions throughout the ten-year course. Patient identifying data have been anonymized throughout this manuscript. All dates and clinical events are accurately reported. The corresponding author holds signed consent documentation on file.

## Structured review

It is not clear to me what this document should contain nor is it specified in the guide for authors.

## Generative AI

Generative artificial intelligence was used for proofreading, and grammar- and spell-checking manually written text using the large language models ChatGPT (GPT 5.6 Sol) and Claude AI (Claude Sonnet 5). The authors take full legal and academic responsibility for the accuracy of the final text.

## Declaration of competing interest

There are no conflicts of interest to report.
