# Supplementary material for: Neurosurgical management of progressive adhesive arachnoiditis with ascending myelopathy following obstetric epidural Anesthesia: An illustrative case with ten-year follow-up and review of the literature
Source: Brain Spine. 2026 Nov 4;6:107494. doi: 10.1016/j.bas.2026.107494 (PMC13760084; doi:10.1016/j.bas.2026.107494)
Supplement: Supplementary file 1 [file mmc1.docx]

**CARE Checklist – Completed**

*Manuscript: Neurosurgical Management of Progressive Adhesive Arachnoiditis with Ascending Myelopathy Following Obstetric Epidural Anaesthesia: An Illustrative Case with Ten-Year Follow-Up and Review of the Literature*

Authors: Veldeman M, Hoellig A, Huffmann B, Clusmann H | Journal: Brain and Spine

| **Topic** | **Item** | **Checklist item description** | **Reported on Line / Location** |
| --- | --- | --- | --- |
| **Title** | 1 | The diagnosis or intervention of primary focus followed by the words “case report” | Title – present. Title reads: “Neurosurgical Management of Progressive Adhesive Arachnoiditis with Ascending Myelopathy Following Obstetric Epidural Anaesthesia: An Illustrative Case with Ten-Year Follow-Up and Review of the Literature” |
| **Key Words** | 2 | 2 to 5 key words that identify diagnoses or interventions in this case report, including “case report” | Keywords listed after abstract: adhesive arachnoiditis; spinal arachnoiditis; epidural anaesthesia; obstetric; ascending myelopathy; syringomyelia; ventriculoperitoneal shunt; cystoperitoneal shunt; neurosurgical management. Note: “case report” not explicitly listed as a keyword – consider adding. |
| **Abstract (no references)** | 3a | Introduction: What is unique about this case and what does it add to the scientific literature? | Abstract – Background section. States this is the longest documented follow-up of post-epidural CAA and the first case documenting progression to brainstem involvement with respiratory compromise. |
|  | 3b | Main symptoms and/or important clinical findings | Abstract – Observations section. Documents progressive ascending myelopathy from paraparesis to near-complete tetraplegia, bulbar symptoms, respiratory failure over 10 years. |
|  | 3c | The main diagnoses, therapeutic interventions, and outcomes | Abstract – Observations section. CAA with ascending myelopathy; VP shunt, hemilaminectomy, CP shunt, Rickham reservoir, suboccipital decompression, dual shunt, tracheostomy; near-complete tetraplegia with preserved cognition. |
|  | 3d | Conclusion — What is the main “take-away” lesson(s) from this case? | Abstract – Lessons section. Each surgical decision must target stabilisation rather than cure; long-term surveillance of full neuraxis is warranted; disease can progress beyond any previously reported timeframe. |
| **Introduction** | 4 | One or two paragraphs summarizing why this case is unique (may include references) | Introduction section (2 paragraphs with references 1–6). Contextualises rarity of post-epidural CAA; states this is the longest follow-up and first case with brainstem progression. |
| **Patient Information** | 5a | De-identified patient specific information | Illustrative Case section. Patient described as “a woman in her mid-twenties”; no name, date of birth, or identifying details disclosed. |
|  | 5b | Primary concerns and symptoms of the patient | Index event subsection: immediate shooting leg pain at epidural placement; incomplete leg recovery; headache, apathy, nausea 2 months later; subsequent progressive paraparesis, hand weakness, bulbar symptoms, respiratory failure. |
|  | 5c | Medical, family, and psycho-social history including relevant genetic information | Stated as “no relevant medical history”. No family or psychosocial history documented – not applicable given acute iatrogenic trigger. Patient’s personal motivation (son) mentioned in Discussion. |
|  | 5d | Relevant past interventions with outcomes | No prior interventions before index event. Complete chronology of all neurosurgical interventions with indications and outcomes provided in Table 1. |
| **Clinical Findings** | 6 | Describe significant physical examination (PE) and important clinical findings | Illustrative Case sections: neurophysiology showing afferent disturbances; MRC grading of leg and arm weakness documented at each stage; facial palsy House-Brackmann 4/5; complex oculomotor paresis; bulbar dysarthria; bilateral vocal cord palsy. |
| **Timeline** | 7 | Historical and current information from this episode of care organized as a timeline | Table 1 provides a complete chronological record of all 17 neurosurgical interventions from Month 2 (Year 1) to Year 9, with time points, indications, and immediate outcomes. Case narrative also structured chronologically by year. |
| **Diagnostic Assessment** | 8a | Diagnostic testing (such as PE, laboratory testing, imaging, surveys) | MRI findings described and referenced (Figs. 1–3) at each disease stage. CSF sterility confirmed. Neurophysiology results reported. Phoniatric and PEG evaluation mentioned. |
|  | 8b | Diagnostic challenges (such as access to testing, financial, or cultural) | Discussed in Discussion: the specific epidural injectate was never documented at the referring institution. This represents a key diagnostic limitation acknowledged by the authors. |
|  | 8c | Diagnosis (including other diagnoses considered) | Primary diagnosis: chronic adhesive arachnoiditis post-epidural anaesthesia, complicated by communicating hydrocephalus, spinal cord compression, syringomyelia, and ascending myelopathy to brainstem. Differential (bacterial meningitis as independent or contributing trigger) discussed. |
|  | 8d | Prognosis (such as staging in oncology) where applicable | Discussed in Discussion and Lessons: prognosis framed as uncertain; progression cannot be excluded even during apparent stability; goal of surgery is stabilisation rather than cure. |
| **Therapeutic Intervention** | 9a | Types of therapeutic intervention (such as pharmacologic, surgical, preventive, self-care) | All 17 surgical interventions listed in Table 1: EVD, VP shunt, hemilaminectomy, CP shunt, Rickham reservoir, suboccipital decompression + C1 resection + duraplasty, dual-valve shunt system, syrinx catheter, PEG, tracheostomy. Antibiotic treatment (vancomycin, meropenem) at referring hospital mentioned. |
|  | 9b | Administration of therapeutic intervention (such as dosage, strength, duration) | Shunt valve types and pressure settings documented (Codman Hakim Medos 140 mmH₂O; Medos Hakim 200 mmH₂O; Certas Plus levels 5 and 2). Surgical levels and catheter positions specified throughout. |
|  | 9c | Changes in therapeutic intervention (with rationale) | Changes documented throughout with explicit rationale: CP shunt exchange for insufficiency; additional valve for overdrainage; shunt ligation for intracranial hypotension; escalation to dual-valve system for unfavourable pressure distribution; integration of 4th ventricle and syrinx drainage (Table 1). |
| **Follow-up and Outcomes** | 10a | Clinician and patient-assessed outcomes (if available) | Clinician-assessed outcomes documented at each stage (MRC scores, MRI regression/progression, clinical symptoms). Patient’s perspective on goals of care (presence for her son) documented. Formal patient-reported outcome measure not used. |
|  | 10b | Important follow-up diagnostic and other test results | Serial MRI results described and illustrated at years 1, 3, 5, 7, and 9–10 (Figs. 1–3). Most recent MRI (>10 years post-index) described in “Current status” section. |
|  | 10c | Intervention adherence and tolerability (How was this assessed?) | Tolerability addressed implicitly: each intervention’s immediate outcome reported; complications (e.g., intracranial hypotension from overdrainage, iatrogenic cerebellar haemorrhage during Rickham aspiration) documented with management. |
|  | 10d | Adverse and unanticipated events | Explicitly reported: iatrogenic cerebellar parenchymal haemorrhage during Rickham reservoir aspiration (Year 9), requiring emergency craniotomy and EVD; CSF fistula from wound disconnection (Year 5). Both managed and documented in Table 1. |
| **Discussion** | 11a | A scientific discussion of the strengths AND limitations associated with this case report | Discussion section addresses limitations: unknown injectate identity; uncertain causal attribution (meningitis vs. epidural); inability to prove surgical benefit definitively. Strengths: 10-year follow-up, comprehensive surgical detail, literature comparison. |
|  | 11b | Discussion of the relevant medical literature with references | Discussion section (refs. 1–6): structured comparison with Boiardi et al. 1983, Killeen et al. 2012, AlMutairi et al. 2024, and Table 2 compares all published surgically managed cases. |
|  | 11c | The scientific rationale for any conclusions (including assessment of possible causes) | Pathophysiological mechanism discussed: CSF flow obstruction by ascending adhesions generating pressure gradients → compartmentalised cysts → syringomyelia → 4th ventricular obstruction → brainstem compression. Aetiological hypotheses (chemical, hyperergic, infectious) reviewed. |
|  | 11d | The primary “take-away” lessons of this case report (without references) in a one paragraph conclusion | Lessons section (structured with three bold subheadings): surgery delays but does not halt progression; patient-centred goals must guide each decision; ascending CAA is a distinct under-described entity requiring full-neuraxis surveillance. |
| **Patient Perspective** | 12 | The patient should share their perspective in one to two paragraphs on the treatment(s) they received | Not present as a standalone section. The patient’s perspective is woven into the narrative (expressed consent for tracheostomy; motivation to survive for her son). Consider adding a brief formal “Patient Perspective” paragraph to meet CARE criteria. |
| **Informed Consent** | 13 | Did the patient give informed consent? Please provide if requested | YES – Patient Consent section states: written consent obtained from patient’s parents (legal next-of-kin); patient was cognitively intact and involved in all treatment decisions; signed consent held by corresponding author. Ticked: Yes. |

*Note: One item requires attention before submission – Item 12 (Patient Perspective): consider adding a brief standalone paragraph reflecting the patient’s own perspective on her treatment. All other items are present in the manuscript.*
